# The Effect of Octopamine on the Locust Stomatogastric Nervous System

**DOI:** 10.3389/fphys.2012.00288

**Published:** 2012-07-20

**Authors:** David Rand, Daniel Knebel, Amir Ayali

**Affiliations:** ^1^Department of Zoology, Faculty of Life Sciences, Tel Aviv UniversityTel Aviv, Israel; ^2^The Sagol School of Neuroscience, Tel Aviv UniversityTel Aviv, Israel

**Keywords:** locust, stomatogastric nervous system, central pattern generators, octopamine, DUM neurons

## Abstract

Octopamine (OA) is a prominent neuromodulator of invertebrate nervous systems, influencing multiple physiological processes. Among its many roles in insects are the initiation and maintenance of various rhythmic behaviors. Here, the neuromodulatory effects of OA on the components of the locust stomatogastric nervous system were studied, and one putative source of OA modulation of the system was identified. Bath application of OA was found to abolish the endogenous rhythmic output of the fully isolated frontal ganglion (FG), while stimulating motor activity of the fully isolated hypocerebral ganglion (HG). OA also induced rhythmic movements in a foregut preparation with intact HG innervation. Complex dose-dependent effects of OA on interconnected FG-HG preparations were seen: 10^−5^ M OA accelerated the rhythmic activity of both the HG and FG in a synchronized manner, while 10^−4^ M OA decreased both rhythms. Intracellular stimulation of an identified octopaminergic dorsal unpaired median neuron in the subesophageal ganglion was found to exert a similar effect on the FG motor output as that of OA application. Our findings suggest a mechanism of regulation of insect gut patterns and feeding-related behavior during stress and times of high energy demand.

## Introduction

**Figure 1:**
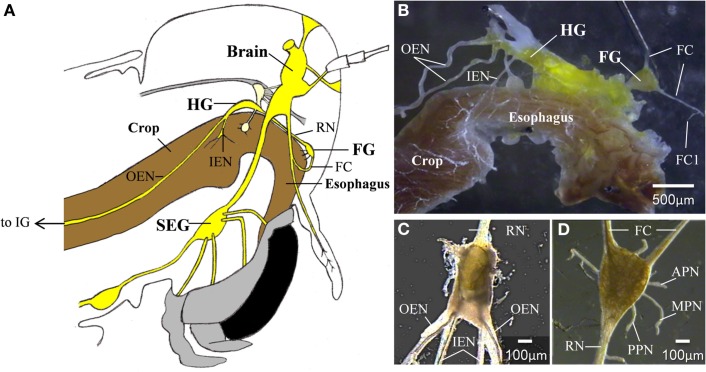
**(A)** A schematic lateral view of a locust head. The relative position of the foregut, central,- and stomatogastric nervous systems can be seen **(B)** The foregut and the STNS. **(C)** The hypocerebral ganglion. **(D)** The frontal ganglion. APN, anterior pharyngeal nerve; FC, frontal connectives; FC1, frontal connective 1; FG, frontal ganglion; HG, hypocerebral ganglion; IEN, inner esophageal nerve; IG, ingluvial ganglion; MPN, median pharyngeal nerve; OEN, outer esophageal nerve; PPN, posterior pharyngeal nerve; RN, recurrent nerve; SEG, subesophageal ganglion.

Locust foregut movements are responsible for passing food particles from the pharynx to the crop, and also for pumping air to inflate the alimentary canal at early stages of the molt (Hughes, 1980; Ayali, 2004, 2009; Zilberstein et al., 2006). Although the gut has some intrinsic contractile capabilities (Oldfield and Huddart, 1982; Banner et al., 1987), foregut movements are under the control of the stomatogastric nervous system (STNS), which is comprised of a small set of peripheral ganglia (Figure 1): the frontal ganglion (FG), the hypocerebral ganglion (HG), and the paired ingluvial ganglia (IG; Chapman, 1998). Anteriorly, the FG is connected to the brain by the paired frontal connectives (FC), which give rise to small branches that innervate the most anterior region of the foregut. An additional three lateral pairs of FG nerves (the anterior, median, and posterior nerves; APN, MPN, and PPN, respectively) branch onto the dilator muscles of the pharynx in a rostrum-to-caudal manner (Allum, 1973; Aubele and Klemm, 1977; Ayali et al., 2002; Ayali, 2004). The FG is connected to the HG via the recurrent nerve (RN). Two pairs of lateral nerves arise from the HG: the inner esophageal nerves (IEN), which innervate the more posterior muscles of the esophagus and the anterior part of the crop; and the outer esophageal nerves (OEN), which branch onto more posterior parts of the crop and terminate in the paired IG. Both the FG and HG function as central pattern generators (CPGs), capable of generating rhythmic motor patterns independent of sensory inputs. These patterns were found to be correlated with foregut movements (Ayali et al., 2002; Rand and Ayali, 2010).

The rate of food passage through the gut is regulated by positive and negative inputs that depend on the physiological state of the insect, food composition, and food volume (Bernays, 1985). Many chemical compounds, primarily biogenic amines and peptides, are known to regulate gut motility. The action of such compounds can directly influence the myogenic properties of the gut (Huddart and Oldfield, 1982; Banner et al., 1987), or modulate the motor outputs of the CPGs that control it (Zilberstein et al., 2004, 2006). One of the most prominent biogenic amines in the nervous system of invertebrates is octopamine (OA), which is known to act as a neurotransmitter, neuromodulator, and hormone. Based on physiological, chemical, and molecular similarities, OA is considered to be the invertebrates homolog of vertebrates nor adrenaline (NA). Both NA and OA are released in times of high energy demands, affecting almost every physiological and behavioral processes in the animal (for review see Roeder, 1999; Verlinden et al., 2010).

In insects, OA is released from a small number of identified neurons, among them are the dorsal unpaired median (DUM) neurons, which are found in all the ganglia of the ventral nerve cord, except from the brain (Bräunig and Pflüger, 2001). It is now well established that DUM neurons project both centrally, innervating neuropiles of different ganglia (Bräunig, 1991), and peripherally, innervating skeletal or visceral musculature (Bräunig, 1990, 1997; Bräunig et al., 1994). The modulatory action of OA on the central nervous system of *Schistocerca americana* was studied by Sombati and Hoyle (1984), by delivering it iontophoretically into distinct regions of the metathoracic neuropil. They found that applied OA evokes and maintains repetitive motor activities of different rhythmic behaviors according to the site of injection. The evoked motor activities were flexion-extension movements of the tibia, flight activity, and ongoing rhythmic oviposition. Furthermore, by depolarization of a single DUM neuron in the metathoracic ganglia they could elicit rhythmic flexion-extension of the tibia, similar to that induced by local OA delivery (Sombati and Hoyle, 1984).

About 250 neurons from the brain and subesophageal ganglion (SEG) were reported to innervate the stomatogastric nervous systems of *Locusta migratoria* via the frontal connectives (Bräunig, 2008). Among these are three identified DUM neurons, two of which, termed DUM SAp 1 and 2, appear to terminate in the FG. They also send axon collaterals to the peripheral nerves of the brain, including the tritocerebral ventral nerve, which innervates the pharyngeal dilator muscles. The third neuron, DUM SAp 7, projects to the FC exclusively and its peripheral target is still unknown (Bräunig, 1990, 1991, 1997).

Very little is known about the effect of OA on the STNS of insects. We have previously suggested that bath application of OA disrupts the spontaneous rhythmic activity of the isolated FG (Zilberstein et al., 2004). In the current study we further investigated the role of OA in modulating the motor patterns associated with the FG and HG of the desert locust, *Schistocerca gregaria*. We tested the dose-dependent effect of OA on the fully isolated FG and HG, as well as on the isolated, interconnected FG + HG preparation. We show that stimulating a single DUM neuron in the SEG in an *in vitro* preparation that includes the brain, SEG, and FG, induces similar effects as OA bath application.

## Materials and Methods

### Animals

All experiments were performed on adult male desert locusts (*S. gregaria*, Forskal), taken from our crowded laboratory culture (Ayali et al., 2002) within the first 2 weeks after the final molt. Prior to dissection, locusts were briefly anesthetized in CO_2_ and according to the required experimental preparation, the brain, foregut, and stomatogastric ganglia were removed, pinned dorsal side up in a transparent Sylgard plate (Sylgard 182 silicon Elastomer, Dow Corning Corp., Midland, MI, USA), and covered with locust saline.

### Saline and chemicals

Locust saline was composed of 147 mM NaCl, 10 mM KCl, 4 mM CaCl_2_, 3 mM NaOH, 10 mM HEPES, pH 7.2–7.4 (Penzlin, 1985). Hypotonic saline was used for intracellular recording: 140 mM NaCl, 10 mM KCl, 2 mM CaCl_2_, 4 mM NaH_2_PO_4_, 6 mM Na_2_HPO_4_ (Clements and May, 1974). All salts were obtained from Frutarom Ltd. (Haifa, Israel). Octopamine (Sigma-Aldrich, Rehovot, Israel) was freshly prepared at 100× stock solution in normal saline, according to the noted final concentrations (i.e., stock = 10^−4^, 10^−3^, and 10^−2^ M for final concentrations of 10^−6^, 10^−5^, and 10^−4^ M, respectively). Fifty microliters of stock solution were added to a final bath volume of 5 ml using an Eppendorf pipette, and dispersed and mixed by gentle agitation of the bath solution using the pipette. Recording were made for 30 min before (control) and after application of octopamine, as well as for at least 30 min after washout.

### Electrophysiology and neurobiotin injection

Foregut movements were monitored using a force transducer (Harvard APP Ltd., Holliston, MA, USA), attached to the foregut wall. For extracellular recordings, the cut endings of the peripheral nerves of the FG and HG (see Figure 1) were gently sucked into the tips of glass pipette electrodes (A-M systems, Sequim, WA, USA). As previously shown (Ayali et al., 2002), in the *in vitro* FG, fully synchronous motor patterns can be recorded from the bilateral pairs of nerves (FC1, APN, MPN, and PPN). These show consistent phase relations under the influence of different modulators (Zilberstein et al., 2004, 2006). Thus, in different experiments we monitored the *in vitro* FG rhythm by the nerve that showed the best recorded signal. Data acquisition was done using a four-channel differential amplifier (Model 1700, A-M Systems), played back in real time, and stored on the computer using Axon Digidata 1440A A-D board (Molecular Devices, Sunnyvale, CA, USA) and AxoScope software (Molecular Devices). For intracellular recordings, the somata of SEG neurons were impaled with a microelectrode filled with 3 M Potassium acetate (DC resistances of 50–70 MΩ), and 4% neurobiotin (Vector Laboratories, Inc., Burlingame, CA, USA) at its tip. To aid in penetrating the ganglionic sheath, a few crystals of type XIV protease (Sigma-Aldrich) were placed on the dorsal side of the SEG for 40 s. Intracellular recordings and stimulation were done using Axoclamp-2B (Molecular Devices). Master 8 (A.M.P.I, Jerusalem, Israel) was used for external triggering. Real time playback and storage was done as described above for the extracellular recordings. All recorded data were analyzed using DataView software (W.J. Heitler, University of St. Andrew, UK).

### Neuroanatomy

Neurobiotin injections were made by way of depolarizing current pulses (2 nA, 500 ms, 1 Hz) delivered for 20–30 min. Following injection, dye was allowed to diffuse within the cells for 1 h at room temperature. Ganglia were fixed in 4% paraformaldehyde in 0.1 M phosphate-buffer saline (PBS, pH 7.0) for overnight at 4°C. They were then rinsed three times at room temperature with 0.1 M PBS, and an additional three times in 0.1M PBS containing 0.1% Triton X-100 (30 min between all rinses). Following overnight incubation with Dylight 488-Streptavidin (Jackson ImmunoResearch Labs, West Grove, PA, USA) in 0.1 M PBS containing 0.3% Triton X-100 (Sigma-Adrich, St. Louis, MO, USA) and rinsing with 0.1 M PBS, ganglia were dehydrated in an ethanol series, and cleared in methyl salicylate (Merck KGaA, Darmstadt, Germany). To visualize injected neurons, ganglia were mounted onto polylysine-coated glass slides under DPX (Fluka) and examined using a ZEISS LSM 510 confocal microscope (Carl Zeiss, Jena, Germany). Horizontal optical sections were taken through each ganglion and the resultant z-series was then projected as a flat image. The dye-filled neurons were analyzed using LSM 5 Image Browser (Carl Zeiss, Jena, Germany).

The significance of results was tested as follows: instantaneous burst frequencies were averaged in 1 min bins. Student’s *t*-test was used to compare the last 10 bins (minutes) in control conditions and 10 bins after OA treatment, starting 2 min after drug application. When possible, frequencies were averaged and compared using one-way analysis of variance (ANOVA), followed by Tukey post-test. Statistical analyses were performed in GraphPad Prism (GraphPad software Inc., San Diego, CA, USA).

## Results

### The effect of octopamine on the *in vitro* isolated FG

**Figure 2:**
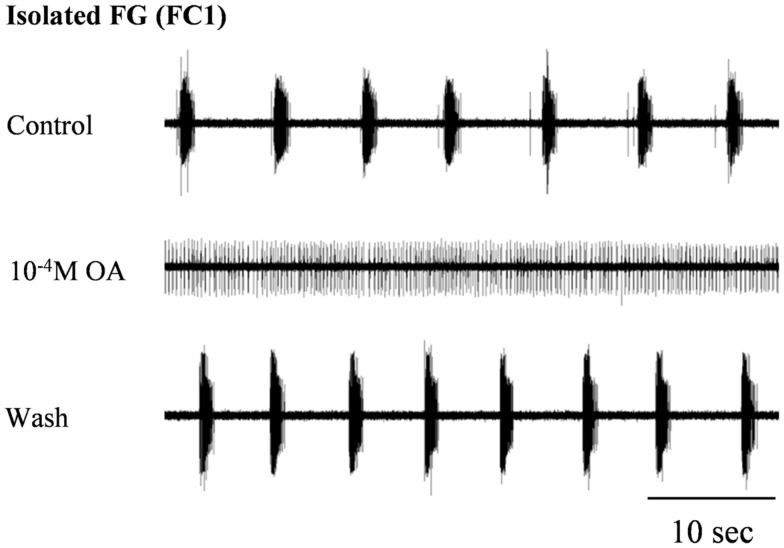
**The disruptive effect of octopamine on the rhythmic activity of the *in vitro* isolated FG**. Extracellular recording from the FG efferent nerve, FC1, before and during bath application of 10^−4^ M OA as well as after wash. OA application resulted in the replacement of HG rhythmic activity by tonic firing.

In order to confirm our previous results on the effect of OA on spontaneous rhythmic patterns of the *in vitro* isolated FG (Zilberstein et al., 2004) we first tested different OA doses (10^−6^, 10^−5^, and 10^−4^ M) by bath application. Octopamine was found to abolish the FG rhythmic motor pattern in a dose-dependent manner: whereas 10^−6^M OA had no effect on the ongoing burst frequency (*n* = 3), application of 10^−5^ and 10^−4^ M OA resulted in complete disappearance of bursting activity in 54% (7 out of 13), and 82% (9 out of 11) of the preparations, respectively. Regardless of the dose, the effect occurred immediately after application and was characterized by replacement of the bursting activity with strong tonic firing (Figure 2). An additional dose-dependent effect was demonstrated by the time it took for rhythmicity to be restored. In four out of the seven preparations that were affected by 10^−5^M OA, rhythmicity was spontaneously restored prior to wash: 11.4 ± 2.8 min after application. In the other three preparations, as in all those that were exposed to 10^−4^ M OA, complete cessation of bursting activity lasted as long as the ganglia were bathed in OA (for at least 30 min) and rhythmicity was only restored after washing with fresh saline. Interestingly, preparations that were still bursting after OA application did not show any significant change in burst frequency (*t*-test comparing burst frequencies before and after drug application – see Materials and Methods).

### The effect of octopamine on the *in vitro* isolated HG

**Figure 3:**
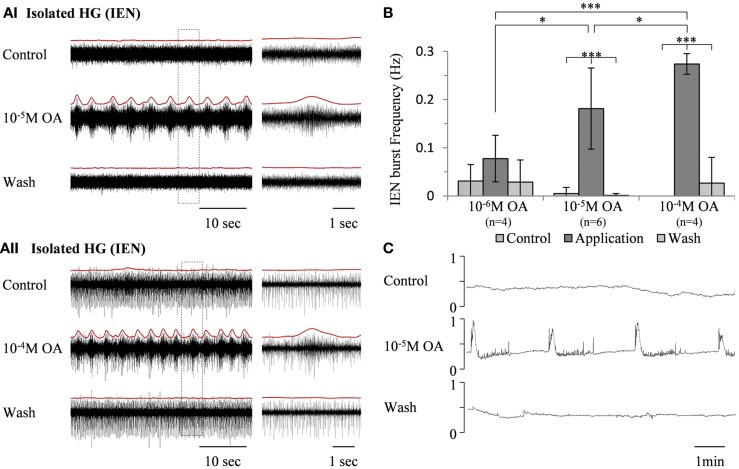
**The excitatory effect of OA on bursting activity of the *in vitro* isolated HG**. **(A)** Extracellular recording from the HG efferent inner esophageal nerve (IEN), before and during bath application of 10^−5^ M OA **(AI)** and 10^−4^ M OA **(AII)**, as well as after wash. Rectified and smoothed signals are shown in red, aligned on top of the unprocessed signal. **(B)** Summary of the dose-dependent excitatory effect. Values are means ± S.D. **(C)** Output of a force transducer connected to the crop wall showing the effect of 10^−5^ M octopamine on an *in vitro* foregut preparation that included the HG, with intact IEN foregut innervation. *y*-Axes are normalized units of the force transducer voltage output.

In contrast to its effect on the rhythmic pattern of the isolated FG, bath application of the same OA concentrations (10^−6^ M, *n* = 4, 10^−5^ M, *n* = 6, and 10^−4^ M, *n* = 4) resulted in dose-dependent excitatory effects on the rhythmic activity recorded from the IEN in *in vitro* isolated HG preparations (Figure 3A). OA either increased burst frequencies in preparations that were spontaneously bursting during control, or generated rhythmic patterns in those that were either silent or firing in a tonic manner. These excitatory effects were completely reversed by washing with fresh saline. One-way ANOVA revealed dose-dependent differences in the HG burst frequencies (*p* < 0.0001). A *post hoc* test (Tukey) is summarized in Figure 3B.

An *in vitro* isolated Gut + HG preparation was studied in order to understand how OA modulation of the bursting pattern of the isolated HG affects gut movements. The foregut was dissected out, leaving the HG on its dorsal side with intact connections of both IENs, while the rest of the STNS ganglia were excluded. Movements were monitored by a force transducer attached to the anterior wall of the crop. As can be seen in Figure 3C, robust rhythmic foregut contractions were generated in response to bath application of 10^−5^ M OA.

### The effect of OA on the interactions of the HG and FG

**Figure 4:**
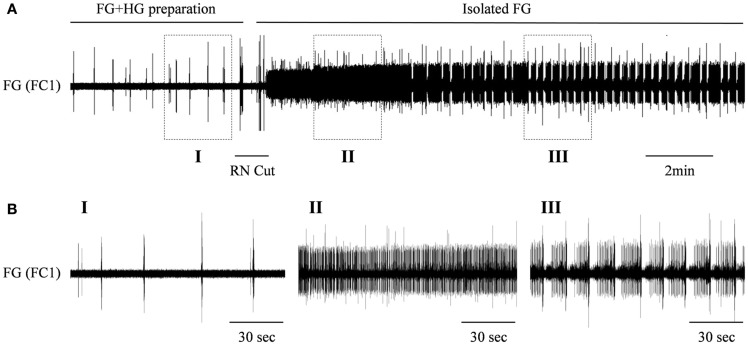
**The effect of the HG on the rhythmic burst frequency of the FG**. **(A)** Upper trace: extracellular recording from the FG efferent nerve, FC1, in an *in vitro* preparation of interconnected FG + HG. RN cut (indicated by a horizontal line) resulted in dense tonic firing of the FG, which later developed into high frequency of bursting activity. **(B)** The outlined frames marked in **(A)** shown on an extended time scale.

We have previously shown a preliminary recording from an *in vitro* isolated preparation that included both the FG and HG (FG + HG preparation) with intact recurrent nerve (RN; Rand and Ayali, 2010). In such a preparation, the HG show two types of bursting patterns, both can be recorded from the IEN: a rhythm that can also be seen in the fully isolated HG (independent HG rhythm: as shown in Figure 3), and an additional rhythm that is synchronized with the activity pattern recorded from the FG (FG-HG rhythm). The latter disappeared when the two ganglia were disconnected by cutting the RN. In order to establish the effect of inputs from the HG on the FG rhythmic activity, *in vitro* isolated FG + HG preparations were studied (*n* = 6) and the RN was cut 30 min after the recordings started. Disconnecting the two ganglia resulted in immediate strong tonic firing of the FG, which 3.4 ± 2.3 min later developed into rhythmic bursting activity (Figure 4). The FG burst frequency was always higher than that seen prior to cutting the RN. The magnitude of the change in activity varied considerably; from 1.3 to 19 times its frequency while still connected to the HG.

**Figure 5:**
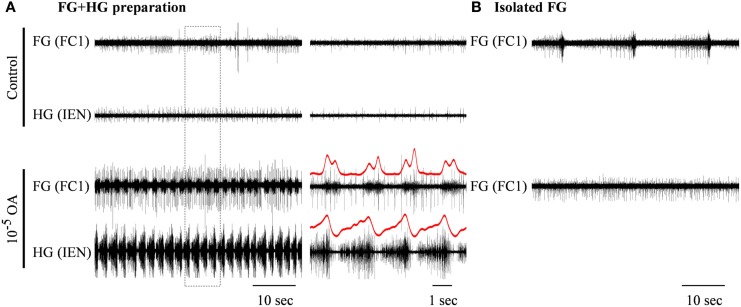
**The excitatory effect of 10^−5^ M octopamine on the rhythmic bursting activity in an *in vitro* FG + HG preparation**. **(A)** Simultaneous extracellular recordings from an FG and an HG nerve. **(B)** Recording from a totally isolated FG placed in the same dish. OA application resulted in the generation of high frequency synchronized bursts in the FG + HG preparation, while the spontaneous bursting activity of the isolated FG disappeared. The outlined frame in the left panel in **(A)** is shown on an extended time scale in the middle panel. FG-HG phase relations can be seen in the red rectified and smoothed signals, which are aligned on top of the unprocessed signal on the extended time scale.

The effects of the two OA doses that were found to be very potent in modulating the isolated HG were also tested on the FG + HG preparation. Bath application of 10^−5^ M OA (*n* = 6) resulted in the appearance of a fully synchronized HG-FG rhythm (in cases where the preparation was silent in control) or in a considerable acceleration of the rhythm if it was slowly bursting in control (with average frequency of 0.073 ± 0.087 Hz). Figure 5 shows one example which included, in the same dish, an FG + HG preparation along side a fully isolated FG. During control, normal FG activity was seen in the fully isolated ganglion, while the FG in the FG + HG preparation was silent. OA application resulted in the appearance of high frequency FG-HG rhythm, while at the same time the rhythmic activity of the fully isolated FG ganglion was replaced by tonic firing. Washing with fresh saline completely reversed the effects of the OA application (not shown).

**Figure 6:**
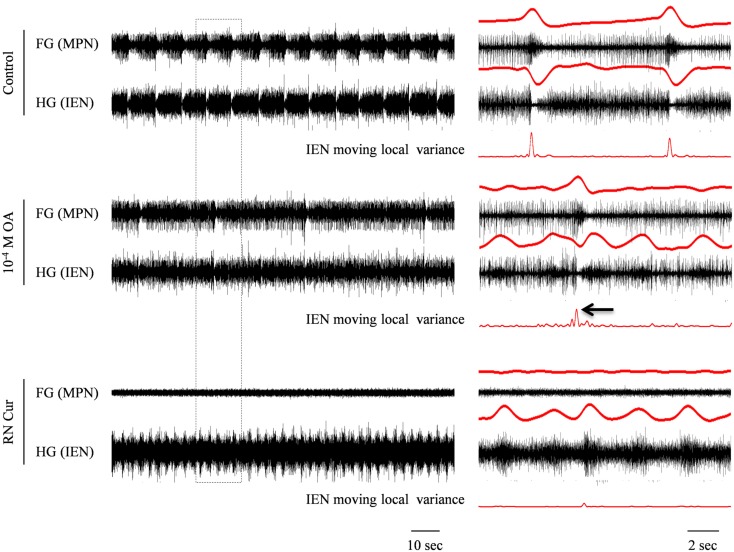
**Simultaneous extracellular recordings from the FG and HG in an *in vitro* FG + HG preparation, showing the effect of 10^−4^ M OA on the FG and HG rhythmic activity**. The outlined frame in the left panel is shown to the right on an extended time scale. The rectified and smoothed signals (red) are aligned on top of the unprocessed signals. OA application resulted in decreased frequency of the synchronized FG + HG bursts and in the appearance of independent HG activity. While the latter is clearly seen in the IEN smoothed signal, the synchronized bursts can still be detected by applying a moving local variance filter. Cutting the RN resulted in complete cessation of the synchronized rhythm seen in both the FG and HG, without affecting the independent HG frequency.

In contrast to the effect of 10^−5^ M OA, application of 10^−4^ M OA (*n* = 3) resulted in reduced frequency of the synchronized HG-FG rhythm. Figure 6 shows an example in which 10^−4^ M OA was applied on to a connected FG + HG preparation that exhibited a spontaneous rhythm during control. The independent HG rhythm, which was either inactive or impossible to trace during the high-frequency synchronized HG-FG rhythmic activity, became apparent on the IEN recording when the HG-FG rhythm decreased under the influence of 10^−4^ M OA. Thirty minutes after OA application the RN was cut, while the preparation was still bathed in OA. Cutting the RN resulted in disappearance of the rhythmic activity of the FG, as well as the HG-FG rhythm from the IEN recording. Now only the HG independent rhythm could be clearly seen.

### DUM neuron stimulation

**Figure 7:**
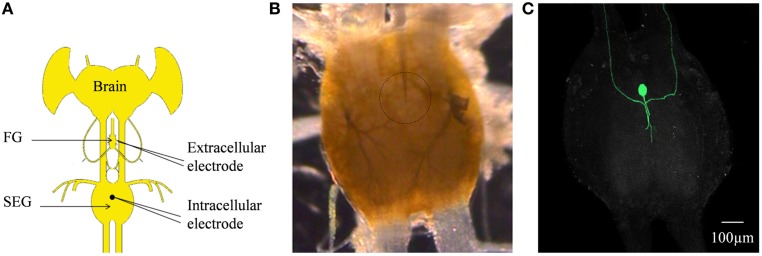
**(A)** Schematic configuration of the Brain + FG + SEG preparation used for the intracellular DUM neuron stimulation and the simultaneous extracellular FG efferent nerve recording. **(B)** Binocular image of The SEG in a preparation as shown in **(C)**. The intracellular electrode can be seen. The position of the neuron is circled. **(C)** Confocal image of the cell shown in **(B)** after neurobiotin injection.

**Figure 8:**
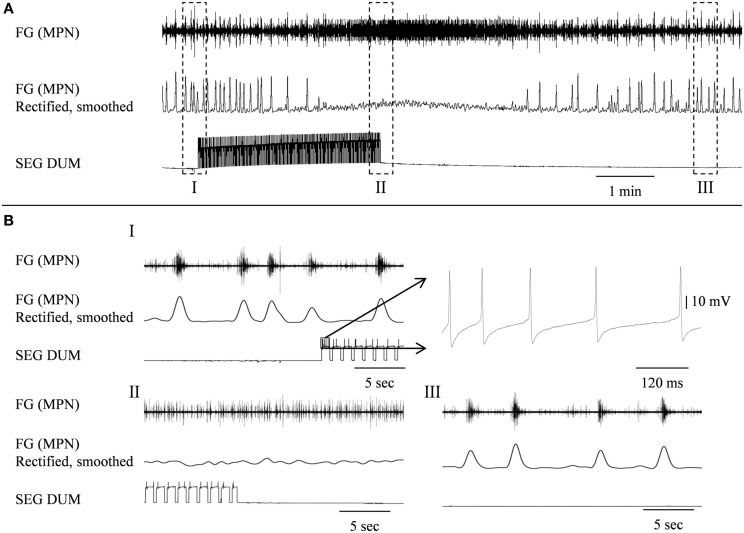
**Stimulating the SEGDUM neuron shown in Figure 7C (Brain + FG + SEG preparation), resulted in prolonged replacement of the FG rhythmic activity by tonic firing**. **(A)** Simultaneous extracellular recording from the FG efferent MPN (upper trace), and intracellular recording from the SEGDUM neuron (lower trace). The smoothed and rectified signal of the MPN is shown in the middle trace. **(B)** Extended time scale of the outlined frames in **(A)**. **(BI)** Rhythmic activity of the FG and the beginning of current injection. The outlined action potentials of the DUM neuron are shown to the right on an extended time scale. **(BII)** Tonic firing of the FG and the end of current injection. **(BIII)** Restoration of rhythmic activity of the FG.

**Figure 9:**
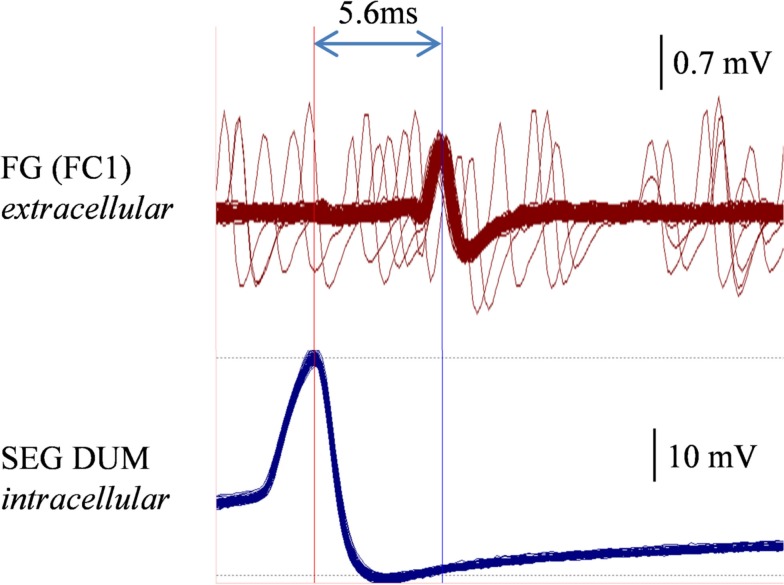
**Overlaying multiple sweeps recorded a preparation similar to the one shown in Figures 7 and 8, while stimulating the same DUM neuron**. Stimulation resulted in a similar disruptive effect on the FG. Sweeps were triggered by the consecutive action potentials of the stimulated DUM neuron (lower trace, left vertical line). A corresponding spike in the extracellular recording of the FG FC1 nerve is clearly seen (upper trace, right vertical line).

In order to determine whether DUM neurons from the SEG constitute a potential source of the observed OA modulation of the STNS motor patterns, *in vitro* preparations were investigated, comprising the brain, SEG, and FG, with intact circumesophageal and frontal connectives (Brain + SEG + FG, see Figure 7A). Neurons at the dorsal midline of the SEG were penetrated with an intracellular pipette electrode and stimulated by current injection, while simultaneously recording from FG efferent nerves. These neurons were viewed later with confocal microscopy, and images were compared with previously described DUM neurons that innervate the STNS. The DUM neuron that is shown in Figure 7C was stimulated in three different preparations at 1 Hz with a duty cycle of 750 ms. In all cases an inhibitory effect on the FG burst frequency was seen, characterized by a slight decrease in burst frequency or complete cessation of the rhythm. Figure 8 shows one example where the SEG DUM neuron stimulation resulted in a prolonged shift from bursting activity of the FG to strong tonic firing. Moreover, we were able to correspond the action potential of this DUM neuron to a single spike recorded from the FG efferent nerve, FC1. This was done by overlaying multiple sweeps triggered by the evoked activity of the DUM neuron to reveal an FG spike firing at a constant lag of 5.6 ms (Figure 9).

## Discussion

In this study we demonstrate that OA has opposite modulatory effects on two principal components of the locust STNS, when isolated from descending and sensory inputs: abolishing the rhythmic bursting activity of the FG and stimulating the rhythmic bursting activity of the HG. While full disappearance of FG rhythm was seen only under the influence of high OA concentrations, the HG was found to be more sensitive to lower concentrations and its increased rhythmic activity was found to be dose-dependent.

Excitation of the HG rhythmic activity was also evident from the induced contractions of the isolated foregut, with intact HG innervation. Huddart and Oldfield (1982) tested the effect of OA (10^−8^–10^−4^ M) on spontaneous contraction of the isolated fore- and hindgut of *L. migratoria*. A dose of 10^−5^–10^−4^ M, OA was found to increase the frequency but diminished the amplitude of the foregut rhythmic contraction (Huddart and Oldfield, 1982). However, unlike in *L. migratoria*, the isolated gut of *S. gregaria* shows little or no spontaneous contractile activity (Banner et al., 1987), and bath application of OA (10^−7^–10^−5^ M) resulted in dose-dependent relaxation (Banner et al., 1989). Therefore, we conclude that our reported foregut contractions are the result of HG stimulation and not a direct effect of OA on the gut.

When the CPGs of the FG and HG are coupled by way of the intact RN, the rhythmic activity of the FG is significantly slower than that seen in the isolated FG after disconnection from the HG (as can be seen in Figure 4 upon cutting the RN). This implies that the HG has an inhibitory effect on the CPG of the FG. The high variability and the extent of the increase in FG frequencies recorded after the RN cut could result from differences in the internal modulatory state of the FG, from differences in the magnitude of HG inhibition, or both. We have previously described the rhythmic activities of the FG (Ayali et al., 2002; Zilberstein and Ayali, 2002) and HG (Rand and Ayali, 2010). When isolated, both ganglia have variable spontaneous frequencies, probably due to their internal modulatory state prior to isolation, i.e., according to the feeding state of the intact animal. In addition to OA, various biogenic amines and neuropeptides are known to modulate the rhythmic activity of the FG (Zilberstein et al., 2004, 2006). It is well established that as well as altering the intrinsic properties of individual neurons, neuromodulators can also alter the strength of synaptic connectivity (for review see Calabrese, 1998; Marder and Thirumalai, 2002). The physiological significance of the HG effect on the FG can be explained by the necessity to cease esophagus contractions when the crop becomes full. This requires sensory inputs from the crop to the HG (Pszolla, Rand and Ayali, unpublished observations), and is integral to the CPG network coupling.

As can be seen in our connected FG + HG preparation, the effect of OA on the coupled ganglia is bi-modal: the bursting rhythmic output of the FG is first accelerated, at the low OA concentrations, and reduced at 10^−4^M. Since acceleration of bursting activity did not occur in the isolated FG, we conclude that it is an indirect result of HG excitation, or dis-inhibition of the HG inhibitory effect discussed above, or both. A rather similar, bi-modal effect of a modulator on coupled oscillators was shown at the cellular level in the pyloric network of lobsters. A complex interaction between the AB and PD neurons take place under the effect of dopamine (DA), which is both dose- and time-dependent. DA was found to exert opposite effects on the isolated neurons: exciting AB and inhibiting the PD neurons. When the AB and PD neurons are connected, PD responds earlier to high doses of DA, while AB is more sensitive to lower doses and has a delayed response. This result in a complex synchronized activity of the AB-PD sub-network (Ayali and Harris-Warrick, 1999).

Stimulating a single neuron from the SEG in the Brain + SEG + FG preparation resulted in full disappearance of the rhythmic bursting activity of the FG, in a similar way to direct OA bath application on the isolated FG. When stimulated, this neuron revealed large amplitude soma spikes with pronounced undershoots during repolarization, typical to DUM neurons (Bräunig and Pflüger, 2001). Only the soma and main axons were filled in our staining attempts, with no apparent bifurcations of smaller neurites, most likely due to poor diffusion of the neurobiotin. Nevertheless, the position of the soma and the morphology of the main branches resemble those of previously described DUM SAp neurons, which innervate the FG via the frontal connectives (Bräunig, 1990, 1991, 1997, 2008). Moreover, it was previously shown that the SEG DUM neurons (located in the area of our stimulated neuron) contain octopamine-immunoreactive cell bodies (Bräunig, 1991). As can be seen in Figure 8, prolonged activity of the DUM neuron was required in order to have an effect on the rhythmic bursting activity of the FG. This may have resulted from the time it takes for the modulator to reach an effective concentration in the FG neuropile. Once an effect is apparent, the transition from bursting activity to its full cessation is characterized by a fast decrease in burst frequency. Finally, bursting activity was completely replaced by strong tonic firing, as in the case of OA bath application (compare, Figures 2 and 8BII). A single spike was seen in the FG efferent nerve, 5.6 ms after each action potential in the stimulated DUM neuron. Since the axonal conduction velocity of locust neurons at 25°C is about 2 m/s (Gray and Robertson, 1998), this time difference corresponds to a distance of 11.2 mm, which fits well the distance that this axon has to pass from the SEG to the FG efferent nerve. Therefore, the origin of this spike may very well be a branch of the stimulated DUM neuron and not a post-synaptic cell. Moreover, in one of the neuroanatomical descriptions of DUM SAp1 and 2, it was shown that these neurons have axon collaterals that extends to peripheral nerves of the FG and directly innervate the foregut, as well as one set of the pharynx dorsal dilator muscles (Bräunig, 1997).

One possible interpretation of our results might be as follows: at the onset of a high energy demand state, DUM neurons from the SEG begin to release OA into the neuropiles of the STNS. Apart from the FG, DUM SAp1 and 2 also innervate the HG via axon collaterals that cross the nervous corpora cardiaca III (NCC III) and the nervous connection between the HG and the corpora cardiaca (Bräunig, 1997). As OA accumulates within the neuropiles, the HG accelerates, and as long as the concentration is too low for abolishing the bursting activity of the FG, the elevated HG activity causes the FG to accelerate as well (At this point in our experiments the FG-HG synchronized type of bursts were clearly noticeable). Once OA concentration is high enough, the activity of the FG stops and only HG bursts (typical to the isolated HG in our experiments) remain active. Thus, when the animal shifts from a resting state to behaviors that require high energy demands, feeding behavior stops, and the esophagus quickly empties. In the case of a long-term behavioral shift, esophageal movements are completely inhibited. Due to technical constraints, our IEN recordings were made close to the origin of this nerve. More distally, this nerve splits and innervates the wall of the crop, as well as gut dilator muscles. The specific target of each HG burst type and its physiological roles in terms of gut contractions are still unknown.

## Conflict of Interest Statement

The authors declare that the research was conducted in the absence of any commercial or financial relationships that could be construed as a potential conflict of interest.
